# Serial computed tomography angiography-based computational fluid dynamics characterizes reduced near-renal tear exchange in kidneys that develop atrophy after aortic dissection repair

**DOI:** 10.3389/fbioe.2026.1945232

**Published:** 2026-10-29

**Authors:** Bingjing Chen, Shujing Shao, Shuting Gao, Tianming Huang, Yuanming Luo, Xiaoyu Qi, Cheng Wang, Kaiming Zhang, Yiqing Li, Chao Yang

**Affiliations:** 1 Department of Vascular Surgery, Union Hospital, Tongji Medical College, Huazhong University of Science and Technology, Wuhan, China; 2 Boea Wisdom (Hangzhou) Network Technology Co., Ltd., Hangzhou, China; 3 School of Mechanical and Manufacturing Engineering, University of New South Wales, Sydney, NSW, Australia

**Keywords:** aortic dissection, computational fluid dynamics, computed tomography angiography, false lumen, re-entry communication, renal atrophy

## Abstract

**Background:**

Renal atrophy may develop after aortic dissection repair despite an anatomically patent renal artery on computed tomography angiography (CTA). This exploratory study compared serial near-renal aortic hemodynamics between kidneys with and without later renal-volume loss.

**Methods:**

Six index kidneys from six men were studied: three with ≥30% loss of reference volume (atrophy group) and three age-matched comparators. Patient-specific computational fluid dynamics (CFD) models were built from preoperative, early postoperative and late follow-up CTA; within each patient, inlet and outlet (three-element Windkessel) boundary conditions were identical across time points. All 90 true-lumen/false-lumen (TL/FL) communications were characterized by distance from the renal-ostial plane, direction, pressure difference and trans-tear flow. TL/FL relationships were quantified at the renal-ostial plane, and bilateral renal-artery flow was computed directly. Renal volume was measured by three-dimensional CTA segmentation.

**Results:**

All six index kidneys were supplied from the FL before repair. Index-kidney volume changed by −53.0%, −64.1% and −50.6% in the atrophy group and by −13.8%, +0.8% and −10.7% in comparators. No hemodynamic measure separated the groups before repair. From the early postoperative model onward (3–37 days), two aortic-level flow measures were lower in every atrophy kidney than in every comparator: the FL flow-magnitude fraction at the renal-ostial plane (0.03–0.10 *versus* 0.13–0.79) and total peak-systolic trans-communication flow (37.8–45.1 *versus* 61.8–185.3 mL/s). Index renal-artery flow separated the groups only at late follow-up (−29.4%, −32.6% and −63.9% *versus* +5.9%, −7.4% and −3.0%), as did tear-exchange flow directed toward the renal-ostial level (1.9–19.1 *versus* 38.7–130.9 mL/s). FL/TL pressure ratios did not separate the groups at any time point. In one patient-derived geometry, sequential virtual closure of idealized communications redistributed and reversed flow in the remaining communications; index renal-artery flow after the final closure was similar to baseline.

**Conclusion:**

In kidneys that later atrophied, the FL flow contribution at the renal level was lower than in comparators early after repair, before the modeled decrease in renal-artery flow and, in two of three kidneys, before substantial volume loss. Given three kidneys per group and multiple measures, these associations are hypothesis-generating and do not establish causation, renal parenchymal perfusion or a treatment threshold.

## 1 Introduction

Aortic dissection creates a double-lumen circulation in which residual communications between the true lumen (TL) and false lumen (FL) continue to shape regional pressure, flow exchange, and branch-vessel hemodynamics after open, endovascular, or hybrid repair (Hagan et al., 2000; Isselbacher et al., 2022). Renal involvement is clinically important because it may present as acute kidney injury, dynamic malperfusion, or delayed structural injury (Crawford et al., 2016; van Bakel et al., 2022).

Routine surveillance relies on serum creatinine, estimated renal function, and computed tomography angiography (CTA) morphology. These measures are necessary but incomplete for unilateral renal injury: the contralateral kidney can compensate biochemically, and a patent renal artery on CTA does not demonstrate adequate flow delivery. Previous imaging studies have associated renal inflow source and dissection involvement with later renal-volume loss (Tsilimparis et al., 2022; Yu et al., 2022). A recent three-case computational fluid dynamics (CFD) series suggested that serial renal-artery-to-aorta pressure ratios may identify persistent hemodynamic compromise before overt atrophy (Yang et al., 2026).

Residual communications are particularly relevant when a kidney depends on FL or mixed TL/FL supply. Their functional contribution cannot be inferred from anatomical presence alone: exchange may be weak, directed away from the renal level, or reorganized after repair. Experimental and computational studies have shown that communication location, multiplicity, and closure alter FL flow, thrombosis-prone conditions, and downstream branch hemodynamics (Menichini et al., 2018; Armour et al., 2020; Li et al., 2022; Armour et al., 2024).

The present study applied established CFD techniques—including TL/FL exchange quantification, three-element Windkessel outlet coupling, inter-luminal pressure analysis, and virtual tear closure—to serial CTA models linked to a three-dimensionally measured renal-volume outcome (Vignon-Clementel et al., 2006; Menichini et al., 2018; Armour et al., 2020; Li et al., 2022).

The objective of this study was to compare serial near-renal hemodynamics between kidneys that did and did not subsequently develop measurable renal atrophy after aortic dissection repair, with boundary conditions held constant within each patient across time points. The analysis combined three-dimensional renal volumetry, directly computed renal-artery flow, near-renal communication-level exchange, renal-ostial aortic-plane TL/FL relationships, qualitative wall-based maps, and a virtual closure experiment. We examined whether kidneys with late volume loss showed a persistent postoperative reduction in tear-exchange flow directed toward the renal-ostial level and in FL flow contribution, with smaller and more heterogeneous changes in local pressure.

## 2 Materials and methods

### 2.1 Study design and case selection

This retrospective, single-center, exploratory case-comparator study was designed as a descriptive imaging-computational analysis. Candidate cases were identified from the institutional CTA archive. Inclusion required aortic dissection involving the renal-artery level with FL or mixed TL/FL supply to the index kidney, a preoperative CTA, an early postoperative CTA approximately 1 month after repair (actual interval 3–37 days; Table 1), a late follow-up CTA approximately 6 months or later after repair (actual interval 173–1,108 days; Table 1), and image quality sufficient for serial reconstruction of the TL, FL and communications.

**TABLE 1:** Patient-level clinical, operative, renal-functional and imaging characteristics of the six index kidneys.

Variable	RA-1	RA-2	RA-3	Non-RA-1	Non-RA-2	Non-RA-3
Age, years	53	38	61	51	55	46
Sex	Male	Male	Male	Male	Male	Male
Hypertension	Yes	Yes	No	Yes	Yes	Yes
Diabetes/coronary disease	No	No	No	No	No	No
Pre-existing renal disease or dialysis	No	No	No	No	No	No
Symptom duration before admission	7 h	5 days	2 days	10 h	29 h	5 h
Primary diagnosis	Stanford type B AD	Stanford type B AD	Stanford type A^a^	Stanford type B AD	Stanford type B AD (DeBakey III)	Stanford type B AD
Repair	TEVAR + LSA chimney	TEVAR	AAR + TAR + FET	TEVAR + SA reconstruction	Aortography + TEVAR	AAR + TAR + FET^b^
Days from repair to CFD models (pre/early/late)	−5/26/340	−2/3/429	−3/9/173	−8/34/377	−5/37/385	−3/9/173
Day of final volumetric CTA	1,108	429	173	722	385	355
Index kidney, side	Left	Left	Left	Right	Left	Right
Index-kidney inflow source on serial CTA	FL (pre, early); TL + FL at day 340; FL at day 1,108	FL (pre, early); TL + FL at day 429	FL	FL (2 RAs)	FL; flap incompletely separating TL and FL at the ostium	FL (2 RAs)
Index renal artery on preoperative CTA	LRA from FL	LRA from FL; intimal flap extending into LRA	LRA from FL	Both RRA branches from FL	LRA from FL; TL/FL communication at the ostial level	Both RRA branches from FL
Communications within 50 mm of renal-ostial plane, n (pre/early/late)^c^	3/1/1	4/3/6	0/2/2	2/2/2	3/1/2	0/2/2
All reconstructed communications, n (pre/early/late)	7/3/3	6/4/8	2/3/3	10/7/6	4/1/2	7/8/6
False-lumen status, last follow-up	Partial thrombosis, extending	Largely thrombosed, residual channel	Partial thrombosis, extending	Partial thrombosis, extending	Complete thrombosis, TL re-expanded	Partial thrombosis, regressing
Pre-event eGFR^d^	64.1	Not determinable	97.3	92.4	98.2	89.4
Last preoperative eGFR	64.1	21.8	98.7	78.8	98.2	104.6
eGFR at final volumetric time point	64.7	61.1	66.9	92.6	–	88.1
Reference renal volume, mL	193.2	225.1	211.9	186.4	201.4	223.8
Final index-kidney volume change	−53.0%	−64.1%	−50.6%	−13.8%	+0.8%	−10.7%
Final contralateral volume change	+12.9%	+14.8%	−15.3%	−12.5%	+1.5%	−11.0%

AD, aortic dissection; TEVAR, thoracic endovascular aortic repair; LSA, left subclavian artery; SA, subclavian artery; AAR, ascending aortic replacement; TAR, total arch replacement; FET, frozen elephant trunk; RA, renal artery; TL, true lumen; eGFR, in mL/min/1.73 m^2^ (CKD-EPI, 2009).

^a^ RA-3: intramural hematoma of the aortic root–arch with a penetrating ulcer, together with dissection from the distal arch to the left external iliac artery; ascending aortic involvement makes this Stanford type A. the intramural component lay proximal to the analyzed segment and was resected.

^b^ non-RA-3 underwent a staged repair: ascending and total arch replacement on day 0 and the endovascular component on day 1; timings are referenced to the start of the index open repair.

^c^ Counted from the tear-level dataset using centerline distance from the renal-ostial plane. The 90 communications analyzed in Tables 3 and 4 comprise all reconstructed communications along the dissected aorta, of which 38 lie within 50 mm of the renal-ostial plane; median distance of all communications 10.3 cm (range 0–26.8 cm).

^d^ Serum creatinine responds to an acute renal insult with a lag of 24–48 h, so a sample obtained within a few hours of symptom onset approximates pre-event renal function. In RA-1, non-RA-1, and non-RA-3 the first creatinine was drawn within 11 h of symptom onset (in non-RA-1 in the emergency department, before ward admission). In non-RA-2 it was obtained on the day of admission, approximately 1 day after onset (sampling time not recorded), and in RA-3 51 h after onset; both values were normal. In RA-2 the first available sample was drawn 5.3 days after onset, so pre-event renal function cannot be determined.

Renal atrophy was defined as a reduction of at least 30% in index-kidney parenchymal volume relative to the reference volume, measured on the preoperative CTA closest to the index repair. Follow-up intervals are expressed in days from the date of repair. In comparator patients, neither kidney met the renal-atrophy criterion, and the FL-supplied kidney was designated the index kidney.

Three atrophy cases met the serial-reconstruction criteria. Three non-atrophic comparator kidneys were then selected using the same anatomical, imaging, and reconstruction requirements, each individually matched 1:1 to one atrophy case by age within 10 years (matched pairs RA-1/non-RA-1, 53 and 51 years; RA-2/non-RA-3, 38 and 46 years; RA-3/non-RA-2, 61 and 55 years); atrophy cases are labeled RA-1 to RA-3 and comparators non-RA-1 to non-RA-3, numbered within each group without reference to the matched pair. All six patients were male, so sex was identical within each matched pair. The analytic unit was one index kidney per patient (three atrophic and three non-atrophic kidneys). The selection and analytical workflow is summarized in Figure 1.

**FIGURE 1:**
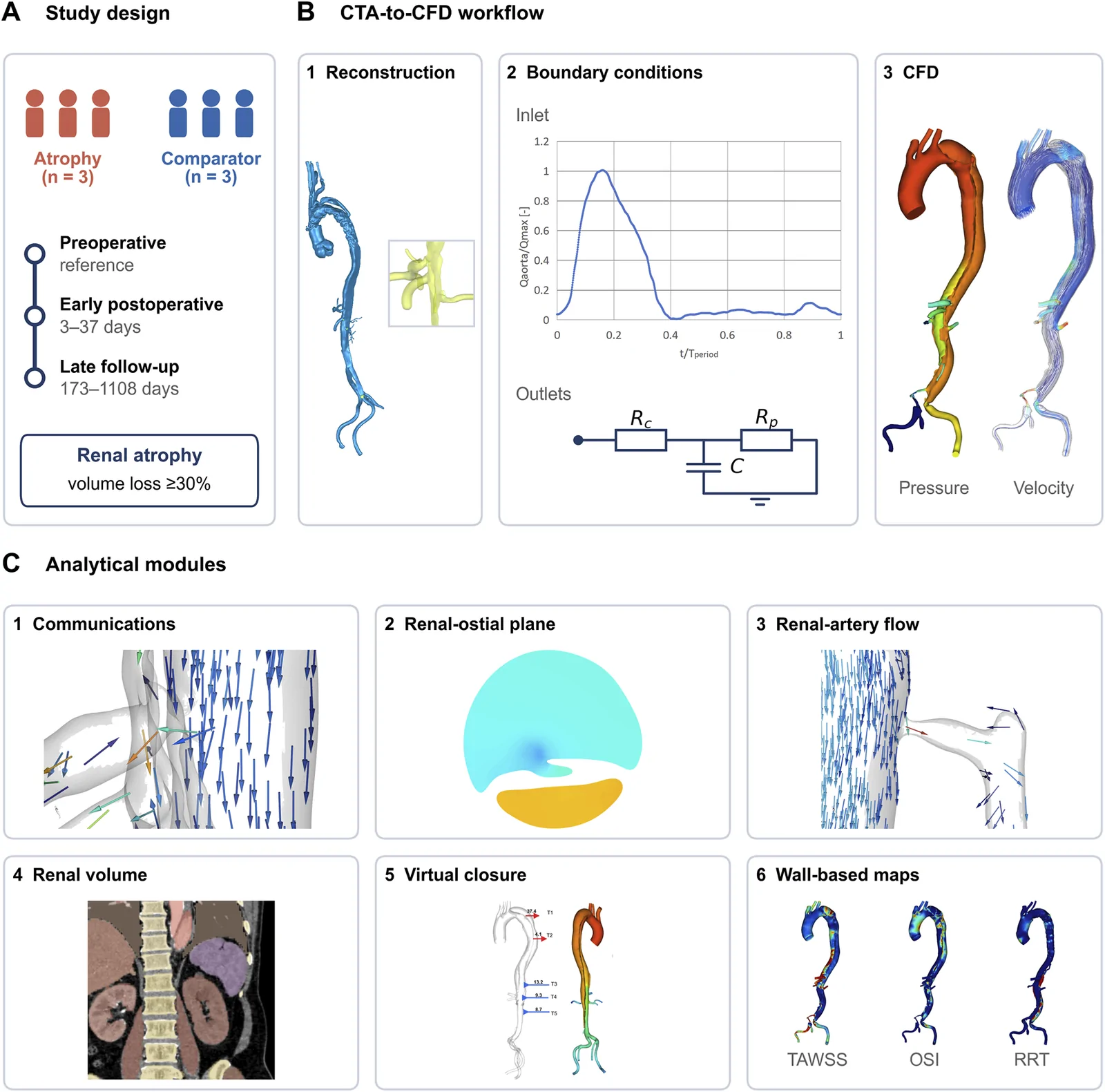
Study design, CTA-to-CFD workflow and analytical modules. **(A)** Exploratory case–comparator design: three index kidneys that developed renal atrophy and three non-atrophic comparator kidneys (six men; comparators matched 1:1 by age within 10 years), each studied at the preoperative CTA closest to repair (reference), an early postoperative CTA (3–37 days) and a late follow-up CTA (173–1,108 days). Renal atrophy was defined as index-kidney volume loss of at least 30% relative to the reference CTA, measured by three-dimensional segmentation. **(B)** Workflow: (1) three-dimensional reconstruction and identification of TL/FL communications (inset, a communication at the renal level); (2) boundary conditions, comprising a published inlet waveform scaled to heart rate and stroke volume (shown normalized, as Qaorta/Qmax against t/Tperiod) and three-element Windkessel outlets (Rc, characteristic impedance; C, compliance; Rp, peripheral resistance) calibrated to clinical blood pressure, identical at all time points within a patient; (3) patient-specific CFD with rigid walls and Newtonian blood (pressure and velocity streamlines are shown). **(C)** Analytical modules: (1) communication-level analysis; (2) TL/FL relationships at the renal-ostial aortic plane; (3) directly computed renal-artery flow; (4) three-dimensional renal volumetry; (5) virtual closure of idealized communications in a patient-derived geometry; (6) qualitative wall-based maps. Representative images are from RA-1, except the whole-aorta reconstruction and the renal segmentation, which are from RA-3.

Patient-level anatomy, repair strategy, renal inflow source, false-lumen status, serial renal function, serial volumetry, and renal-artery flow are reported for each of the six kidneys in Table 1. RA-3 was classified as Stanford type A because the index CTA showed crescentic wall thickening from the aortic sinus to the arch with a penetrating ulcer and a double-lumen configuration extending from the distal arch to the left external iliac artery; the patient underwent ascending and total arch replacement. The intramural component lay proximal to the analyzed peri-renal segment and was resected.

Demographic characteristics, cardiovascular risk factors, blood pressure, repair strategy, renal inflow source, serum creatinine, estimated glomerular filtration rate (eGFR), and blood urea nitrogen were obtained from medical records. This study was approved by the Institutional Review Board/Ethics Committee of Union Hospital, Tongji Medical College, Huazhong University of Science and Technology (approval No. 2024-0393; 16 May 2024). Written informed consent was waived because the study was retrospective and used de-identified clinical and imaging data.

Laboratory times were the sampling times recorded in the hospital information system, not the result-reporting times, and time relative to repair was calculated from the operative start time in the operative record. The complete timed creatinine series for all six patients is provided in Supplementary Table S1.

### 2.2 CTA assessment and renal-volume measurement

CTA examinations were reviewed for renal-artery patency, renal-artery involvement by dissection, index-kidney inflow source, and residual TL/FL communications. Inflow was classified as TL, FL, or mixed TL/FL according to multiplanar CTA review and reconstructed luminal geometry.

Renal parenchymal volume was measured by three-dimensional segmentation of every available CTA using 3D Slicer (version 5.12.4; Fedorov et al., 2012) with the TotalSegmentator automated organ-segmentation extension (Wasserthal et al., 2023). Left and right kidneys were segmented separately (Figure 2A), and all segmentations were reviewed and, where necessary, manually corrected by a single observer. Scans were anonymized and presented in random order, so that the observer was blinded to patient identity, imaging time point, outcome group and CFD results. Percentage volume change was calculated as 100 × (V_follow-up − V_reference)/V_reference.

**FIGURE 2:**
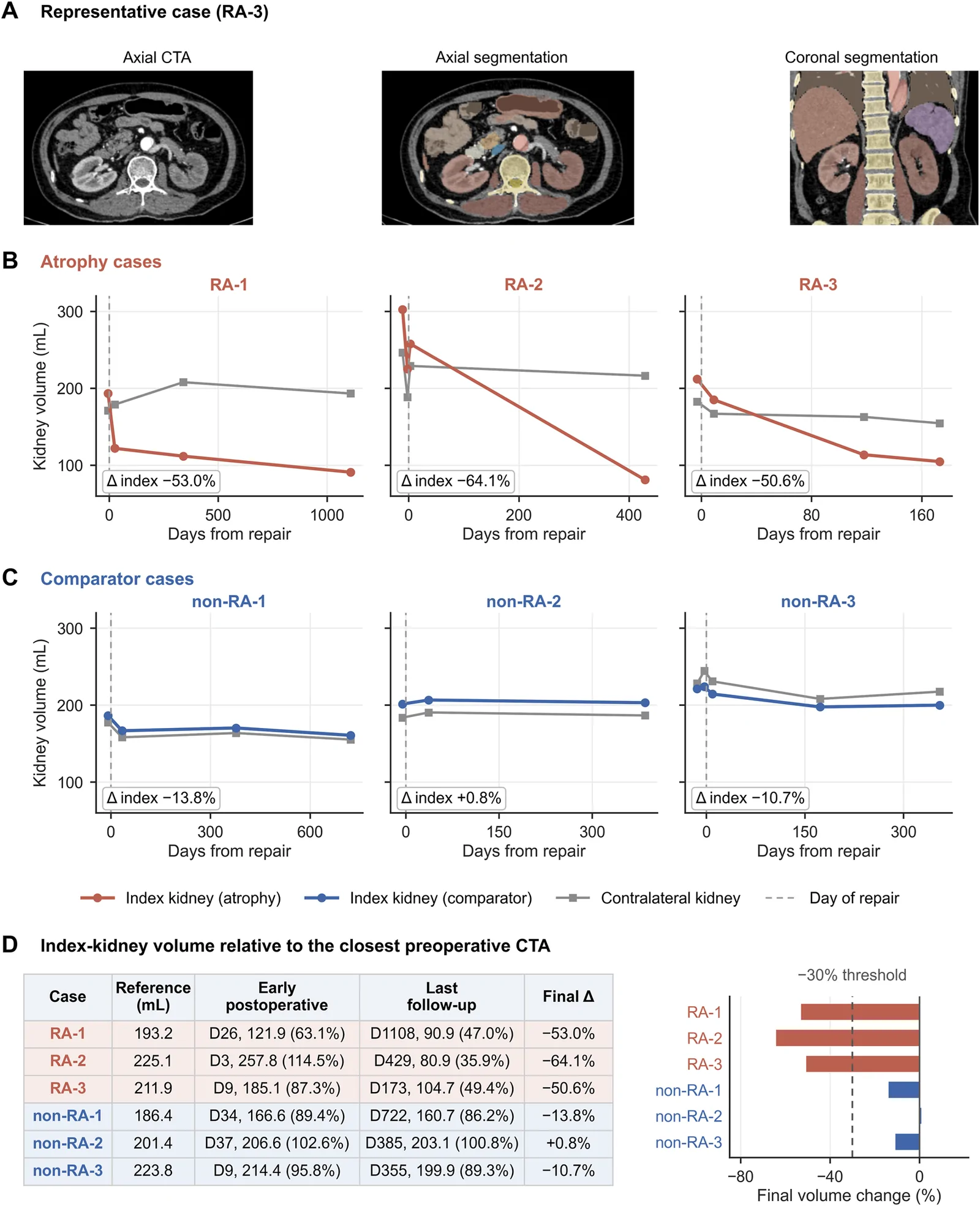
Serial three-dimensional renal volumetry. **(A)** Representative axial CTA with the corresponding axial and coronal TotalSegmentator segmentation (RA-3). **(B)** Serial index-kidney (red) and contralateral (gray) volumes in the three atrophy cases plotted against days from repair; the dashed vertical line marks the day of repair. **(C)** Corresponding trajectories in the three comparator cases (index kidney in blue). All available CTA examinations are plotted, including earlier preoperative scans; percentage change is calculated from the closest preoperative CTA. **(D)** Reference, early postoperative and last-follow-up index-kidney volumes, with final change relative to the closest preoperative CTA; the dashed vertical line marks the −30% renal-atrophy threshold.

### 2.3 Geometry reconstruction and CFD simulation

Patient-specific vascular models were reconstructed from the preoperative, early postoperative, and late follow-up CTA DICOM datasets using DetecModeling software (version 3.2; Boea Wisdom Technology, Hangzhou, China). The reconstructed domain included the thoracoabdominal aorta, TL, FL, celiac trunk, superior mesenteric artery, bilateral renal arteries, aortoiliac outlets, and relevant residual communications. Segmentation used semi-automatic thresholding followed by manual refinement, and smoothing preserved branch ostia, intimal-flap boundaries, and communications.

CFD simulations were performed using DetecFluid software (Boea Wisdom Technology). The domain was discretized using unstructured tetrahedral elements with a maximum element size of 0.75 mm and local refinement around the intimal flap, renal ostia, and communications. The unsteady three-dimensional incompressible Navier–Stokes equations were solved with the finite-volume method. Blood was modeled as a Newtonian fluid with density 1,060 kg/m^3^ and dynamic viscosity 0.004 Pa·s. Vessel walls were assumed rigid, with a no-slip boundary condition. In this study, adaptive time stepping was adopted, with the Courant–Friedrichs–Lewy (CFL) number maintained below 1. Simulations were run for at least three cardiac cycles, and periodic convergence was achieved when the difference in pressures between two consecutive cycles was less than 2 mmHg. Results were extracted from the final cycle.

### 2.4 Boundary conditions

#### 2.4.1 Inlet

The inlet waveform was based on the ascending-aortic flow waveform measured by phase-contrast magnetic resonance imaging in patients undergoing thoracic endovascular aortic repair (Gallo et al., 2016). For each patient, the cardiac period was calculated from the heart rate (HR; T = 60/HR) and the systolic fraction was set from the electrocardiographic timing; the waveform was represented as a Fourier series, and its amplitude was scaled so that the flow integrated over one cardiac cycle equaled the stroke volume measured by transthoracic echocardiography.

#### 2.4.2 Outlets

All branch and distal aortoiliac outlets were coupled to three-element Windkessel (RCR) models, each consisting of a characteristic impedance (Rc), a compliance (C) and a peripheral resistance (Rp) (Vignon-Clementel et al., 2006; Kim et al., 2009; Westerhof et al., 2009). Parameters were obtained in two stages using the approach of Li and Mao (2023). First, a steady-state CFD solution was used to characterize the geometric resistance of each branch. Second, with this geometric resistance fixed, an optimization algorithm searched the RCR parameter space for an equivalent lumped-parameter circulation whose predicted pressures matched the patient’s clinically recorded blood pressure. The calibrated parameters were then used in the unsteady multiscale simulation.

#### 2.4.3 Serial models

Within each patient, the inlet waveform and all outlet Windkessel parameters were identical across the serial models, and no parameter was re-estimated between time points. This design isolates hemodynamic changes associated with vascular geometry. Patients with clinically evident cardiac disease were excluded, and none of the six had a documented cardiac change during follow-up. Gallo et al. (2016) used a similar strategy, applying a single inlet waveform to immediate-postoperative and 1-year models; the main hemodynamic conclusions did not change significantly when an alternative measured waveform or a ±10% perturbation in mean flow was tested.

#### 2.4.4 Renal outlets

The renal outlet Windkessel parameters were estimated once, from the preoperative baseline model, by the procedure above and were not changed at subsequent time points. Renal function, renal volume, and outcome classification were not used in parameter assignment, so simulated renal flow was not explicitly conditioned on the renal-atrophy outcome.

Because four-dimensional flow magnetic resonance imaging (4D-flow MRI), Doppler, or invasive measurements were not available for calibration, absolute CFD-derived flows were treated as model estimates conditional on the prescribed outlet impedance, not as externally validated physiological measurements. The analysis therefore focused on longitudinal changes and between-group contrasts under the same modeling assumptions.

### 2.5 Numerical verification

Periodicity was assessed by comparing systolic and diastolic pressures between consecutive cardiac cycles. A solution was considered periodic when both differed by less than 2 mmHg, and physiologically acceptable when the converged systolic and diastolic pressures were within 10 mmHg of the clinical values used for calibration. Mesh sensitivity was examined in one representative model with maximum element sizes of 0.5, 0.75 and 1 mm (5.519, 2.307 and 1.353 million elements). Relative to the 0.5-mm mesh, the largest instantaneous pressure difference over the cardiac cycle at the inlet and the major-branch outlets was 2.3% (1.9 mmHg) for the 0.75-mm mesh and 2.5% (2.2 mmHg) for the 1-mm mesh; cycle-averaged pressures differed by no more than 1.5% for the 0.75-mm mesh (1.7% for the 1-mm mesh), and the distributions of time-averaged wall shear stress (TAWSS), oscillatory shear index (OSI) and relative residence time (RRT) were visually similar across the three meshes (Supplementary Figures S1 and S2). The 0.75-mm mesh was used for all production simulations. Temporal sensitivity was examined in the same model by comparing fixed time steps of 0.01 s and 0.001 s with CFL-controlled adaptive stepping (CFL = 1); pressure waveforms and wall-based maps obtained with adaptive stepping agreed closely with those obtained with the 0.001-s step (Supplementary Figures S3 and S4).

### 2.6 Near-renal communication analysis

The renal-ostial aortic plane was defined as the cross-section perpendicular to the aortic centerline and passing through the ostium of the index renal artery (Figure 3). All reconstructed TL/FL communications along the dissected aorta were included, and each was located by its centerline distance from this plane. Across the 90 communications, the median distance from the plane was 10.3 cm (range 0–26.8 cm), and 38 lay within 50 mm of it. Per-patient counts under both definitions are given in Table 1.

**FIGURE 3:**
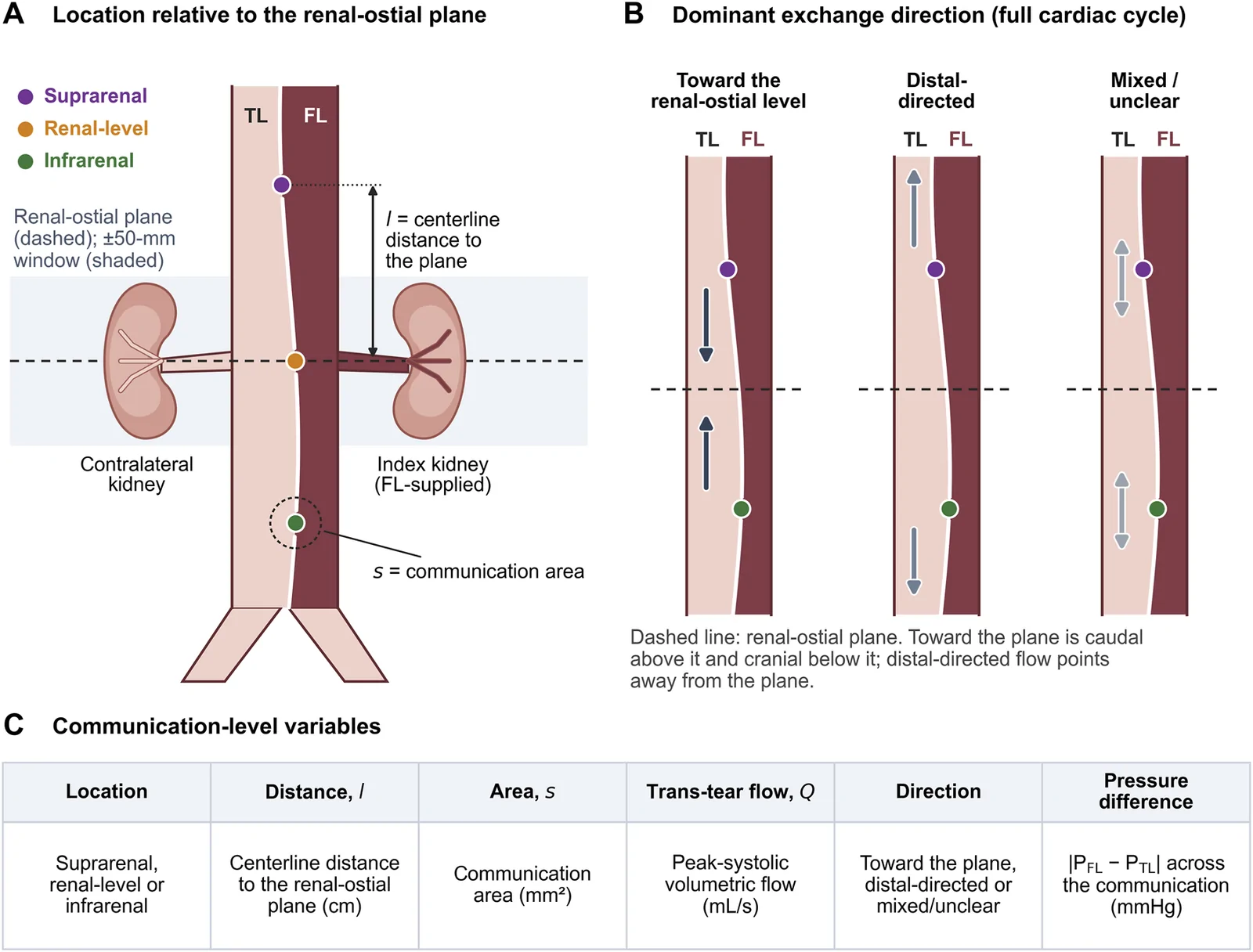
Definition of the renal-ostial aortic plane and communication-level analysis. **(A)** Each reconstructed TL/FL communication was classified as suprarenal, renal-level or infrarenal and located by its centerline distance (l) from the renal-ostial plane, the cross-section through the ostium of the index renal artery; communication area (s) was also measured. The shaded band marks the ±50-mm window used in the sensitivity analysis. **(B)** Dominant exchange direction over the complete cardiac cycle, determined from time-resolved streamlines and classified as directed toward the renal-ostial level, distal-directed, or mixed/unclear. **(C)** Communication-level variables.

All 90 reconstructed communications were included in the primary communication-level analysis, with centerline distance from the renal-ostial plane retained as a continuous descriptive variable. A secondary sensitivity analysis was restricted to the 38 communications within 50 mm of the plane. Communications were classified as proximal (suprarenal), renal-level, or distal (infrarenal); renal-level communications were those whose openings intersected the renal-ostial plane. Variables included centerline distance to the plane, communication area, trans-tear volumetric flow (Q), TL pressure, FL pressure, and the signed and absolute TL–FL pressure differences. Trans-tear flow and the associated TL and FL pressures are peak-systolic values, whereas renal-artery flow (Section 2.8) is cycle-averaged; the two are therefore not numerically comparable.

The dominant exchange direction was determined from time-resolved streamlines over the complete final cardiac cycle. A communication was classified as directed toward the renal-ostial level when the dominant pathway was oriented toward that level, distal-directed when oriented away from it, and mixed/unclear when the pathway was bidirectional, temporally variable, or not reliably classifiable. This variable describes the orientation of flow crossing TL/FL communications within the aorta at the level of the renal ostia; it does not establish that this flow enters a renal artery and is reported separately from the directly computed renal-artery flow (Section 2.8). For communications above the renal-ostial plane, flow directed toward that level corresponds to antegrade (caudal) flow, and for communications below it to retrograde (cranial) flow. Direction was classified by consensus between one author and one CFD engineer, neither of whom was blinded to outcome group. Communication counts were treated as descriptive anatomical observations, not as independent statistical samples.

### 2.7 Renal-ostial aortic-plane hemodynamic analysis

At the renal-ostial aortic plane, TL and FL regions were delineated separately. Peak and cycle-averaged velocity, pressure, and signed volumetric flow were extracted over the final cardiac cycle. Pressure and velocity were summarized as FL/TL ratios. Because the sign of Q depends on the predefined surface-normal direction, local flow contribution was summarized as the FL flow-magnitude fraction, |Q_FL_|/(|Q_FL_| + |Q_TL_|), which is bounded between 0 and 1. This metric describes the distribution of flow magnitude between the two aortic lumina at the renal level and was not interpreted as renal-artery perfusion. Serial values for each kidney are shown in Figure 4.

**FIGURE 4:**
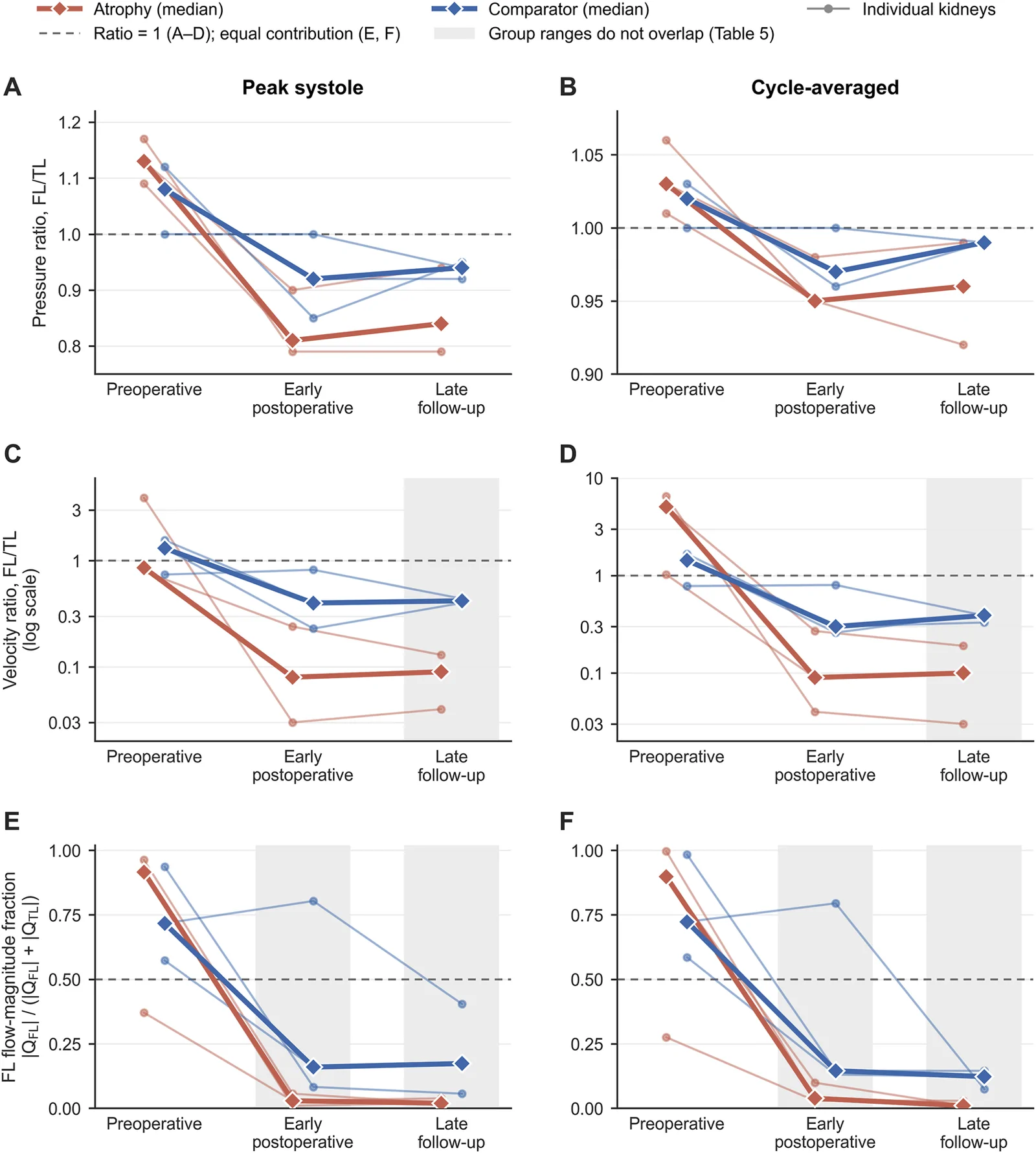
Serial false-lumen/true-lumen (FL/TL) relationships at the renal-ostial aortic plane. **(A)** Peak and **(B)** cycle-averaged pressure ratio; **(C)** peak and **(D)** cycle-averaged velocity ratio; **(E)** peak and **(F)** cycle-averaged false-lumen flow-magnitude fraction, |Q_FL_|/(|Q_FL_|+|Q_TL_|). Velocity ratios **(C,D)** are plotted on a logarithmic axis. Thin lines are individual kidneys and thick diamond-marked lines are group medians. Dashed lines mark a ratio of 1 in **A–D** and equal contribution from the two lumina in **E,F**. Shaded time points indicate non-overlapping ranges between the two groups (Table 5); the groups are offset horizontally for clarity. Flow is reported as the bounded magnitude fraction defined in Section 2.7 rather than as an FL/TL flow ratio, because that ratio is unstable when true-lumen flow approaches zero.

### 2.8 Renal-artery flow and flow redistribution

Renal-artery flow was obtained directly from the computed velocity field. In each model, a cross-section perpendicular to the vessel centerline was defined in the proximal renal artery, and the normal velocity component was integrated over this cross-section to obtain instantaneous volumetric flow. Cycle-averaged values are reported for both renal arteries at each time point. These values are model predictions under the prescribed, fixed outlet impedance and do not represent measured renal parenchymal perfusion. Because the same outlet parameters were used at all CFD time points within a patient, this quantity describes how aortic geometry and tear configuration redistribute flow to the renal arteries under constant downstream impedance.

Because a flow waveform is prescribed at the inlet, external outlet flow over the cardiac cycle is constrained by mass conservation. Flow crossing TL/FL communications is internal to the computational domain and was therefore interpreted as redistribution between luminal pathways rather than as a gain or loss of total flow.

### 2.9 Wall-based indices and virtual closure experiment

TAWSS, OSI, and RRT were visualized in the peri-renal aorta. These maps were used as qualitative supporting evidence of local flow disturbance and low washout, not as validated diagnostic endpoints (Ku et al., 1985; Himburg et al., 2004; Lee et al., 2009).

A virtual perturbation experiment was performed in the preoperative aortic geometry of one atrophy case (RA-1). Five TL/FL communications (T1–T5) were created virtually at prescribed locations, two in the arch and proximal descending aorta and three near the renal-ostial level; they did not correspond to the patient’s native tears. The patient’s native communications were closed in this model. The FL communicated only with the index renal artery, so that, by mass conservation, the cycle-averaged net TL→FL transfer summed across all communications equals the index renal-artery flow. The virtual communications were then sealed sequentially (T1; T1 + T2; T1 + T2 + T3), and the surface was remeshed for each state. The inlet waveform, outlet Windkessel conditions, blood properties, wall assumptions, mesh strategy, and numerical settings were identical across states. The experiment characterized redistribution within the residual TL/FL communication network under fixed computational conditions.

### 2.10 Statistical analysis

All analyses were descriptive and hypothesis-generating. The primary analytic unit was the index kidney. Because each patient contributed an index and a contralateral kidney measured on the same CTA and modeled under the same prescribed boundary conditions, the within-patient difference in change between the two kidneys (ΔΔ) was calculated for renal volume and for cycle-averaged renal-artery flow. This paired contrast reduces the influence of patient-level confounding shared by both kidneys while preserving the asymmetry between them. It was not prespecified and is reported as exploratory. For renal volume, it mainly indicates whether volume loss was unilateral, because the outcome groups are themselves defined by index-kidney volume change; the renal-flow contrast is the informative comparison. To examine the timing of between-group differences, each hemodynamic measure was assessed at each time point for complete separation of the two groups, defined as non-overlapping ranges. With three kidneys per group, the probability of complete separation in a prespecified direction by chance is 1 in 20 for a single comparison. Because 12 measures were examined at three time points, isolated separations were expected by chance; separations were therefore interpreted only when they persisted across time points and occurred in measures derived from independent analyses. Communication-level variables were aggregated within each patient and time point, and group summaries are reported as medians and ranges because each group contained three kidneys. Repeated tear-level and time-point observations are nested within six patients and are not independent samples. Given the sample size and this nesting, no hypothesis testing, correlation analysis, multivariable modeling, or diagnostic threshold estimation was performed, and no correlation coefficients or p values are reported.

## 3 Results

### 3.1 Cohort characteristics and renal-volume endpoints

All six patients were male. Median age was 53 years (range 38–61) in the atrophy group and 51 years (46–55) in comparators. None had diabetes, coronary artery disease or a diagnosis of chronic kidney disease, and none was receiving renal replacement therapy before admission; hypertension, present in five patients, was the only recorded comorbidity. Patient-level characteristics are given in Table 1.

Because serum creatinine rises 24–48 h after an acute renal insult, a sample drawn within a few hours of symptom onset approximates pre-event renal function. In three of the six patients, the first creatinine was obtained within 11 h of symptom onset (at 9.6, 4.5 and 10.5 h), corresponding to pre-event eGFRs of 64.1, 92.4 and 89.4 mL/min/1.73 m^2^, respectively; in one of these comparators, a creatinine value obtained 8 years before the aortic event was also normal. In two further patients, one comparator and one atrophy case, the first sample was obtained approximately 1 day (sampling time not recorded) and 51 h after onset, respectively, and was normal. In the remaining atrophy case, who presented 5 days after symptom onset, pre-event renal function could not be determined; renal function deteriorated further before repair, with eGFR falling from 52.0 to 8.3 mL/min/1.73 m^2^ and serum potassium reaching 6.82 mmol/L, and continuous renal replacement therapy was started. The last preoperative eGFR was 64.1, 21.8 and 98.7 mL/min/1.73 m^2^ in the atrophy group and 78.8, 98.2 and 104.6 mL/min/1.73 m^2^ in comparators.

All six index renal arteries remained anatomically identifiable on serial CTA, and residual TL/FL communications were present in all six patients, whereas only three index kidneys lost volume. Renal-artery patency and residual communications did not distinguish the kidneys that later atrophied. Index-kidney volume fell by 53.0%, 64.1% and 50.6% in the atrophy group, whereas comparator index kidneys changed by −13.8%, +0.8% and −10.7% (Figure 2; Table 1). The contralateral kidney changed by +12.9%, +14.8% and −15.3% in the atrophy group and by −12.5%, +1.5% and −11.0% in comparators; in RA-1 and RA-2 it enlarged, consistent with compensatory hypertrophy, whereas in RA-3 both kidneys lost volume. Expressed as the within-patient difference between the index and the contralateral kidney, volume change was −65.9%, −78.9% and −35.3% in the atrophy group and −1.3%, −0.7% and +0.3% in comparators (Figure 5E); because the groups are defined by index-kidney volume, this contrast documents that the loss was predominantly unilateral and is not an independent result. At the early postoperative CTA (3–37 days after repair), index-kidney volume was 36.9% below reference in RA-1, 14.5% above reference in RA-2 and 12.7% below reference in RA-3, compared with −10.6%, +2.6% and −4.2% in comparators; substantial volume loss was already present in RA-1 but not in RA-2 or RA-3.

**FIGURE 5:**
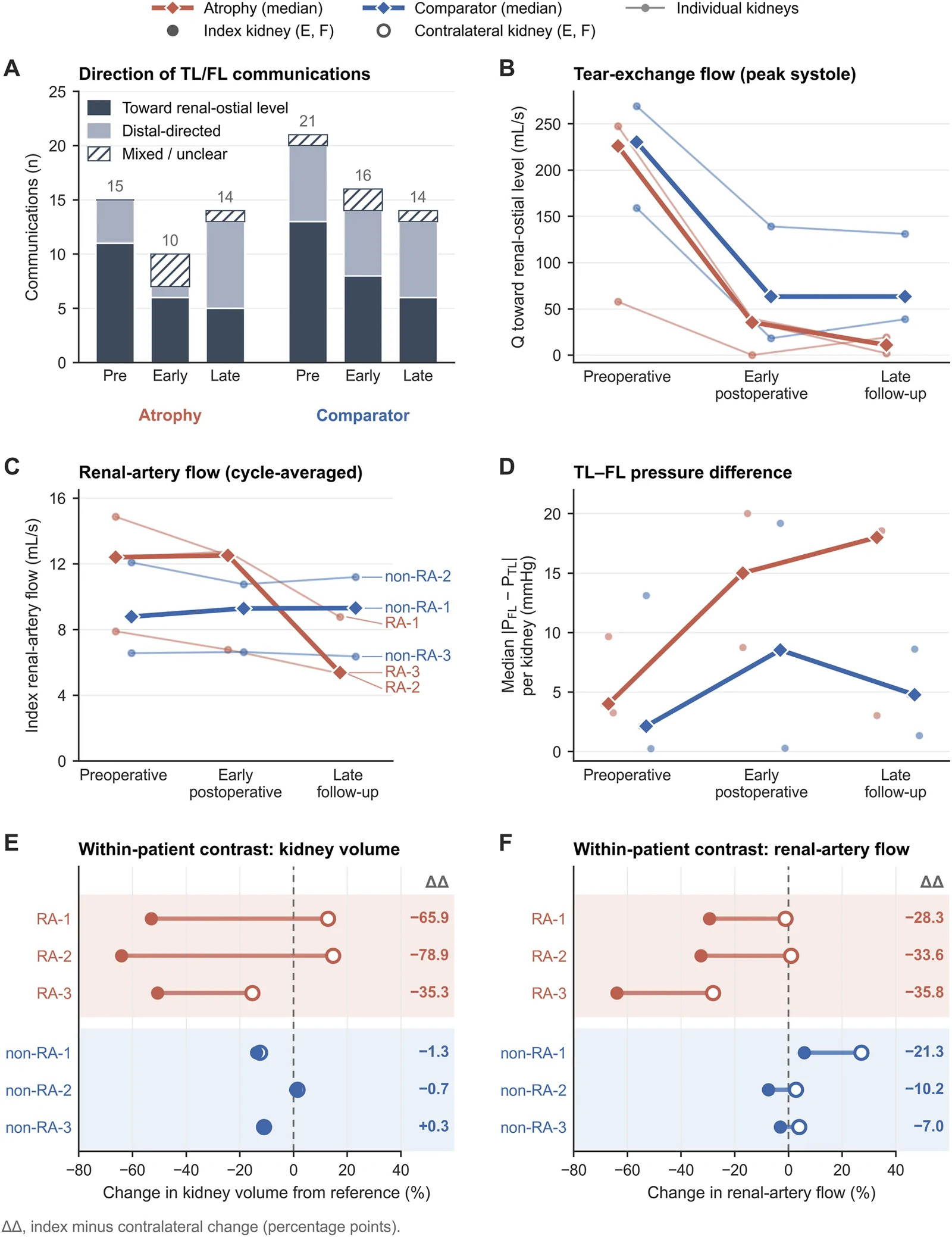
Serial hemodynamic and volumetric findings. **(A)** Full-cycle dominant direction of all 90 TL/FL communications by group and time point. **(B)** Patient-aggregated peak-systolic tear-exchange flow directed toward the renal-ostial level. **(C)** Directly computed cycle-averaged index renal-artery flow. **(D)** Patient median absolute TL–FL pressure difference. **(E)** Within-patient contrast between index and contralateral renal-volume change relative to the closest preoperative CTA. **(F)** Within-patient contrast in change of cycle-averaged renal-artery flow. Thin lines or small points are individual kidneys and thick diamond-marked lines are group medians. ΔΔ, index minus contralateral change in percentage points. Communication-level data were aggregated within each patient and time point before group summary. No correlation or hypothesis testing was performed.

On preoperative CTA, the index kidney was supplied from the FL in all six patients, and reduced parenchymal enhancement was reported in all six index kidneys. During follow-up, the supply of two atrophy kidneys became mixed (RA-1 at day 340 and RA-2 at day 429), and in non-RA-2 the intimal flap did not completely separate the two lumina at the renal ostium (Table 1). FL inflow and reported reduced enhancement did not by themselves distinguish kidneys that subsequently atrophied from those that did not.

### 3.2 Renal-artery flow

Cycle-averaged flow in the index renal artery decreased in all three atrophy cases, by 29.4%, 32.6% and 63.9%, while flow in the contralateral renal artery changed by −1.1%, +1.0% and −28.1%, respectively. Among comparators, index renal-artery flow changed by +5.9%, −7.4% and −3.0%. Expressed as the within-patient index-minus-contralateral difference, flow change was −28.3%, −33.6% and −35.8% in the atrophy group and −21.3%, −10.2% and −7.0% in comparators (Figure 5F). The index-minus-contralateral reduction was larger in every atrophy kidney than in every comparator; the smallest between-group difference was 7.0 percentage points (−28.3% in RA-1 *versus* −21.3% in non-RA-1). Absolute values at every quantified time point are given in Table 2, and serial trajectories are shown in Figure 5C.

**TABLE 2:** Cycle-averaged renal-artery flow (mL/s) derived by direct integration of the normal velocity component.

Case	Index kidney	Preoperative	Early postoperative	Late follow-up	Index change	Contralateral change
RA-1	Left	12.40	12.74	8.76	−29.4%	−1.1%
RA-2	Left	7.89	6.77	5.32	−32.6%	+1.0%
RA-3	Left	14.86	12.51	5.37	−63.9%	−28.1%
Non-RA-1	Right	8.78	9.28	9.30	+5.9%	+27.2%
Non-RA-2	Left	12.08	10.75	11.19	−7.4%	+2.8%
Non-RA-3	Right	6.56	6.63	6.36	−3.0%	+4.0%

Values are model predictions obtained under the prescribed, fixed outlet impedance and do not represent measured renal parenchymal perfusion.

Between the preoperative and early postoperative models, index renal-artery flow changed by +2.7%, −14.2% and −15.8% in the atrophy group and by +5.7%, −11.0% and +1.1% in comparators. Renal-artery flow did not separate the groups at this time point; the reduction in the atrophy group occurred between the early and late models. The earliest between-group differences were at the aortic level (Section 3.5). For RA-1, non-RA-1 and non-RA-3, the late CFD model preceded the final volumetric CTA (day 340 *versus* 1,108, 377 *versus* 722 and 173 *versus* 355 after repair, respectively); in the other three patients, the same CTA was used for both (Table 1).

### 3.3 Serial location and direction of near-renal communications

Ninety communications were identified along the dissected aorta: 39 in the atrophy group and 51 in comparators. Before repair, 11 of 15 atrophy-group communications (73.3%) and 13 of 21 comparator communications (61.9%) were directed toward the renal-ostial level. At the early postoperative time point, the corresponding proportions were 6 of 10 (60.0%) and 8 of 16 (50.0%). At late follow-up, communications directed toward the renal-ostial level decreased to 5 of 14 (35.7%) in the atrophy group and 6 of 14 (42.9%) in comparators, while distal-directed communications accounted for 8 of 14 and 7 of 14, respectively (Table 3; Figure 5A).

**TABLE 3:** Descriptive distribution of communication location and full-cycle flow direction.

Group	Time point	n	Proximal	Renal-level	Distal	Directed toward renal-ostial level	Distal-directed	Mixed/unclear
Atrophy	Preoperative	15	7 (46.7)	3 (20.0)	5 (33.3)	11 (73.3)	4 (26.7)	0
Atrophy	Early postoperative	10	4 (40.0)	2 (20.0)	4 (40.0)	6 (60.0)	1 (10.0)	3 (30.0)
Atrophy	Late follow-up	14	4 (28.6)	4 (28.6)	6 (42.9)	5 (35.7)	8 (57.1)	1 (7.1)
Comparator	Preoperative	21	12 (57.1)	3 (14.3)	6 (28.6)	13 (61.9)	7 (33.3)	1 (4.8)
Comparator	Early postoperative	16	4 (25.0)	4 (25.0)	8 (50.0)	8 (50.0)	6 (37.5)	2 (12.5)
Comparator	Late follow-up	14	3 (21.4)	4 (28.6)	7 (50.0)	6 (42.9)	7 (50.0)	1 (7.1)
Total	—	90	34 (37.8)	20 (22.2)	36 (40.0)	49 (54.4)	33 (36.7)	8 (8.9)

Values are n (% within group and time point). Counts represent anatomical communications and were not treated as independent statistical observations.

The distance of communications from the renal-ostial plane also differed between groups. Median distance at the three time points was 6.6, 4.7 and 4.6 cm in the atrophy group and 14.5, 13.3 and 10.5 cm in comparators. Pooled across time points, the median was 4.7 cm (range 0–26.8) in the atrophy group and 12.1 cm (0–24.2) in comparators, and 22 of 39 atrophy-group communications (56%) lay within 50 mm of the plane compared with 16 of 51 (31%) in comparators. However, one comparator had communications as close to the renal ostium as any atrophy case (per-patient median 2.6, 0.7 and 2.6 cm at the three time points), so proximity alone did not separate the two outcomes in this series.

In the secondary sensitivity analysis restricted to the 38 communications within 50 mm of the renal-ostial plane, median exchange flow directed toward that level was 18.9, 13.6 and 8.1 mL/s in the atrophy group and 7.7, 8.2 and 63.3 mL/s in comparators at the three time points. The late follow-up separation was preserved, whereas the preoperative ordering reversed because two kidneys had no communication within 50 mm before repair. Because spatial restriction changed the composition of the analyzed communication network, this analysis is treated as a localized sensitivity analysis rather than as a replacement for the all-communication analysis.

### 3.4 Patient-aggregated exchange flow and pressure relationships

After patient-level aggregation, the atrophy group showed a larger and more persistent postoperative reduction in tear-exchange flow directed toward the renal-ostial level. Median peak-systolic Q decreased from 225.8 mL/s (range 57.6–247.3) before repair to 35.4 mL/s (0.0–39.3) early after repair and 10.9 mL/s (1.9–19.1) at late follow-up. Comparator values were 230.2 mL/s (159.0–268.9), 63.2 mL/s (18.1–139.0), and 63.3 mL/s (38.7–130.9), respectively (Table 4; Figure 5B). In every patient, the largest preoperative communication directed toward the renal-ostial level lay 12.6–26.8 cm above the plane (area 131–393 mm^2^), indicating that the preoperative values mainly reflect flow through large suprarenal communications that repair was intended to exclude. The decrease from preoperative to early postoperative values occurred in both groups. At late follow-up, communications within 50 mm of the plane carried all of the exchange directed toward the renal-ostial level in five patients and 74% of it in the sixth.

**TABLE 4:** Patient-aggregated communication metrics.

Metric	Group	Preoperative	Early postoperative	Late follow-up
Communications per kidney, n	Atrophy	6 (2–7)	3 (3–4)	3 (3–8)
Communications per kidney, n	Comparator	7 (4–10)	7 (1–8)	6 (2–6)
Total trans-communication Q (peak systolic), mL/s	Atrophy	225.8 (78.3–281.3)	39.3 (37.8–45.1)	33.0 (19.1–39.8)
Total trans-communication Q (peak systolic), mL/s	Comparator	265.4 (244.5–316.5)	63.2 (61.8–185.3)	80.0 (68.3–205.2)
Exchange Q directed toward renal-ostial level (peak systolic), mL/s	Atrophy	225.8 (57.6–247.3)	35.4 (0.0–39.3)	10.9 (1.9–19.1)
Exchange Q directed toward renal-ostial level (peak systolic), mL/s	Comparator	230.2 (159.0–268.9)	63.2 (18.1–139.0)	63.3 (38.7–130.9)
Fraction of total Q directed toward renal-ostial level	Atrophy	0.88 (0.74–1.00)	0.94 (0.00–1.00)	0.48 (0.06–0.57)
Fraction of total Q directed toward renal-ostial level	Comparator	0.85 (0.65–0.87)	0.75 (0.29–1.00)	0.64 (0.48–0.93)
Median |PFL−PTL|, mmHg	Atrophy	4.0 (3.2–9.7)	15.0 (8.7–20.0)	18.0 (3.0–18.6)
Median |PFL−PTL|, mmHg	Comparator	2.1 (0.2–13.1)	8.5 (0.3–19.2)	4.8 (1.3–8.6)

Values are patient-level median (range), n = 3 kidneys per group. Communication-level data were aggregated within each patient and time point before group summaries were calculated.

Total trans-tear exchange was also persistently reduced in the atrophy group. Individual trajectories were heterogeneous, particularly at the early postoperative time point, but all three atrophy kidneys had low exchange flow directed toward the renal-ostial level at late follow-up. The median patient-level absolute TL–FL pressure difference increased from 4.0 to 15.0 and 18.0 mmHg in the atrophy group, compared with 2.1, 8.5, and 4.8 mmHg in comparators (Figure 5D). The reduction in exchange flow was not accompanied by a uniform loss of local pressure transmission. Because the inlet waveform and all outlet impedances were identical across the serial models within each patient, these changes reflect aortic remodeling, progressive FL thrombosis and the residual tear configuration rather than changes in the prescribed boundary conditions.

Because individual communications carried flow in opposite directions, patient-level exchange was also expressed as signed net transport, defined as the sum of distal-directed flow minus the sum of flow directed toward the renal-ostial level, so that a negative value denotes net transport toward that level. Before repair, net transport was negative in all six patients (atrophy −213.3, −225.8 and −36.9 mL/s; comparators −73.5, −194.9 and −247.3 mL/s). At late follow-up, it was approximately zero or reversed in all three atrophy kidneys (−2.8, +21.8 and +1.5 mL/s) and in one comparator (+2.5 mL/s), whereas the other two comparators retained substantial net transport toward the renal-ostial level (−63.3 and −56.7 mL/s). This signed metric separated the groups less completely than the directional counts.

### 3.5 Renal-ostial aortic-plane hemodynamic relationships

Figure 4 shows individual serial trajectories of the cycle-averaged pressure ratio, velocity ratio, and FL flow-magnitude fraction at the renal-ostial aortic plane. In the atrophy group, the median FL/TL pressure ratio was 1.03 preoperatively, 0.95 early after repair and 0.96 at late follow-up, whereas the median FL/TL velocity ratio decreased from 5.06 to 0.09 and 0.10, and the median FL flow-magnitude fraction from 0.90 to 0.04 and 0.01. In comparators, pressure ratios remained near unity; velocity ratios and flow contribution also decreased after repair but generally remained higher than in the atrophy group at late follow-up.

These measurements describe TL/FL hemodynamic relationships within the aortic cross-section at the renal level and are reported separately from the directly computed renal-artery flow (Section 3.2).

Timing of between-group differences. Table 5 gives the range of each hemodynamic measure in each group at each time point. No measure separated the groups before repair. From the early postoperative model onward, two aortic-level flow measures derived from independent analyses were lower in every atrophy kidney than in every comparator: the cycle-averaged FL flow-magnitude fraction at the renal-ostial plane (0.03–0.10 *versus* 0.13–0.79; peak values 0.01–0.06 *versus* 0.08–0.80) and the total peak-systolic trans-communication flow (37.8–45.1 *versus* 61.8–185.3 mL/s). Both remained separated at late follow-up. The FL/TL velocity ratios, exchange flow directed toward the renal-ostial level and index renal-artery flow separated the groups only at late follow-up; the FL/TL pressure ratios and the TL–FL pressure difference did not separate them at any time point. At the early postoperative CTA, index-kidney volume was 14.5% above reference in RA-2 (day 3) and 12.7% below reference in RA-3 (day 9), whereas RA-1 had already lost 36.9% (day 26).

**TABLE 5:** Timing of between-group differences: range of each hemodynamic measure in the atrophy and comparator groups at each time point.

Measure	Preoperative	Early postoperative	Late follow-up
FL/TL peak pressure ratio	1.09–1.17 vs. 1.00–1.12	0.79–0.90 vs. 0.85–1.00	0.79–0.94 vs. 0.92–0.95
FL/TL cycle-averaged pressure ratio	1.01–1.06 vs. 1.00–1.03	0.95–0.98 vs. 0.96–1.00	0.92–0.99 vs. 0.99
FL/TL peak velocity ratio	0.79–3.91 vs. 0.74–1.56	0.03–0.24 vs. 0.23–0.82	0.04–0.13 vs. 0.40–0.44 separated
FL/TL cycle-averaged velocity ratio	1.02–6.50 vs. 0.78–1.66	0.04–0.27 vs. 0.26–0.80	0.03–0.19 vs. 0.33–0.41 separated
Peak FL flow-magnitude fraction	0.37–0.96 vs. 0.57–0.94	0.01–0.06 vs. 0.08–0.80 separated	0.02–0.04 vs. 0.06–0.40 separated
Cycle-averaged FL flow-magnitude fraction	0.28–1.00 vs. 0.59–0.98	0.03–0.10 vs. 0.13–0.79 separated	0.01–0.03 vs. 0.07–0.15 separated
Total trans-communication Q (peak systolic), mL/s	78.3–281.3 vs. 244.5–316.5	37.8–45.1 vs. 61.8–185.3 separated	19.1–39.8 vs. 68.3–205.2 separated
Exchange Q directed toward renal-ostial level (peak systolic), mL/s	57.6–247.3 vs. 159.0–268.9	0.0–39.3 vs. 18.1–139.0	1.9–19.1 vs. 38.7–130.9 separated
As above, communications within 50 mm of the plane, mL/s	0.0–36.0 vs. 0.0–8.7	0.0–21.4 vs. 0.0–63.2	1.9–19.1 vs. 38.7–130.9 separated
Median |PFL−PTL| per kidney, mmHg	3.2–9.7 vs. 0.2–13.1	8.7–20.0 vs. 0.3–19.2	3.0–18.6 vs. 1.3–8.6
Index renal-artery flow (cycle-averaged), mL/s	7.89–14.86 vs. 6.56–12.08	6.77–12.74 vs. 6.63–10.75	5.32–8.76 vs. 6.36–11.19
Change in index renal-artery flow from preoperative, %	–	−15.8 to +2.7 vs. −11.0 to +5.7	−63.9 to −29.4 vs. −7.4 to +5.9 separated

Values are the range (minimum to maximum) across the three kidneys in each group, atrophy *versus* comparator; “separated” indicates non-overlapping ranges. Renal-ostial plane values are FL/TL ratios; FL flow-magnitude fraction = |QFL|/(|QFL| + |QTL|). With three kidneys per group, complete separation in a prespecified direction has a chance probability of 1 in 20 for a single comparison; 12 measures were examined at three time points.

### 3.6 Qualitative wall-based maps

TAWSS, OSI, and RRT maps are shown in Figure 6. Areas of low shear, oscillatory shear, and prolonged residence were visible in peri-renal FL regions in both groups. The maps provide spatial context for local flow disturbance but were not quantified or used to define a group-specific threshold.

**FIGURE 6:**
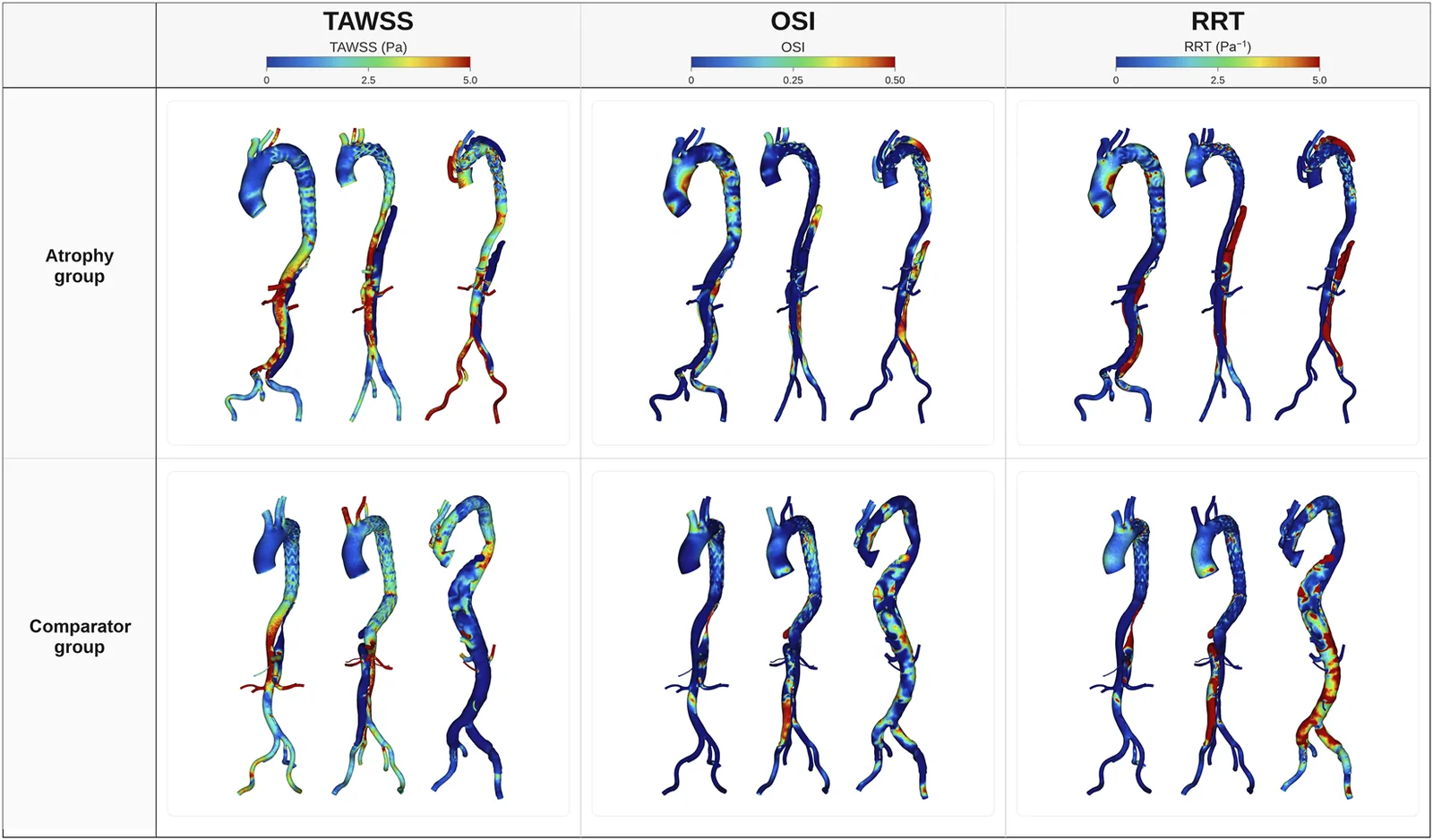
Qualitative wall-based maps. Time-averaged wall shear stress (TAWSS), oscillatory shear index (OSI), and relative residence time (RRT, Pa^−1^) distributions are shown for representative atrophy and comparator models. These maps provide spatial context for disturbed and low-washout peri-renal flow and were not used to define quantitative group thresholds.

### 3.7 Virtual closure of selected suprarenal communications

Results of the virtual perturbation experiment are shown in Figure 7. With boundary conditions and numerical settings held constant, sequential closure reduced FL→TL exchange directed toward the renal-ostial level from 31.3 mL/s at baseline to 9.1, 0.9 and 0.0 mL/s (cycle-averaged values; communications T1–T5 were created virtually, see Section 2.9). This reduction follows by construction, because the sealed communications were removed from the model. In the remaining communications, flow through T2 increased more than four-fold, from 4.1 to 17.6 mL/s, after T1 was sealed. Flow direction then reversed progressively: T3 changed from FL→TL (13.2 mL/s) to TL→FL (9.0 mL/s) after the second closure, and T4 and T5 both reversed after the third. The net inter-luminal transfer summed across all communications changed little across the four states (+10.2, +8.5, +8.1 and +10.5 mL/s, TL→FL positive; summed TL→FL flow minus summed FL→TL flow). Because the FL drained only into the index renal artery, this net transfer equals the index renal-artery flow. After the third closure, index renal-artery flow was similar to baseline (difference 0.3 mL/s); the decrease in directed exchange reflected redistribution within the network rather than loss of flow from the domain.

**FIGURE 7:**
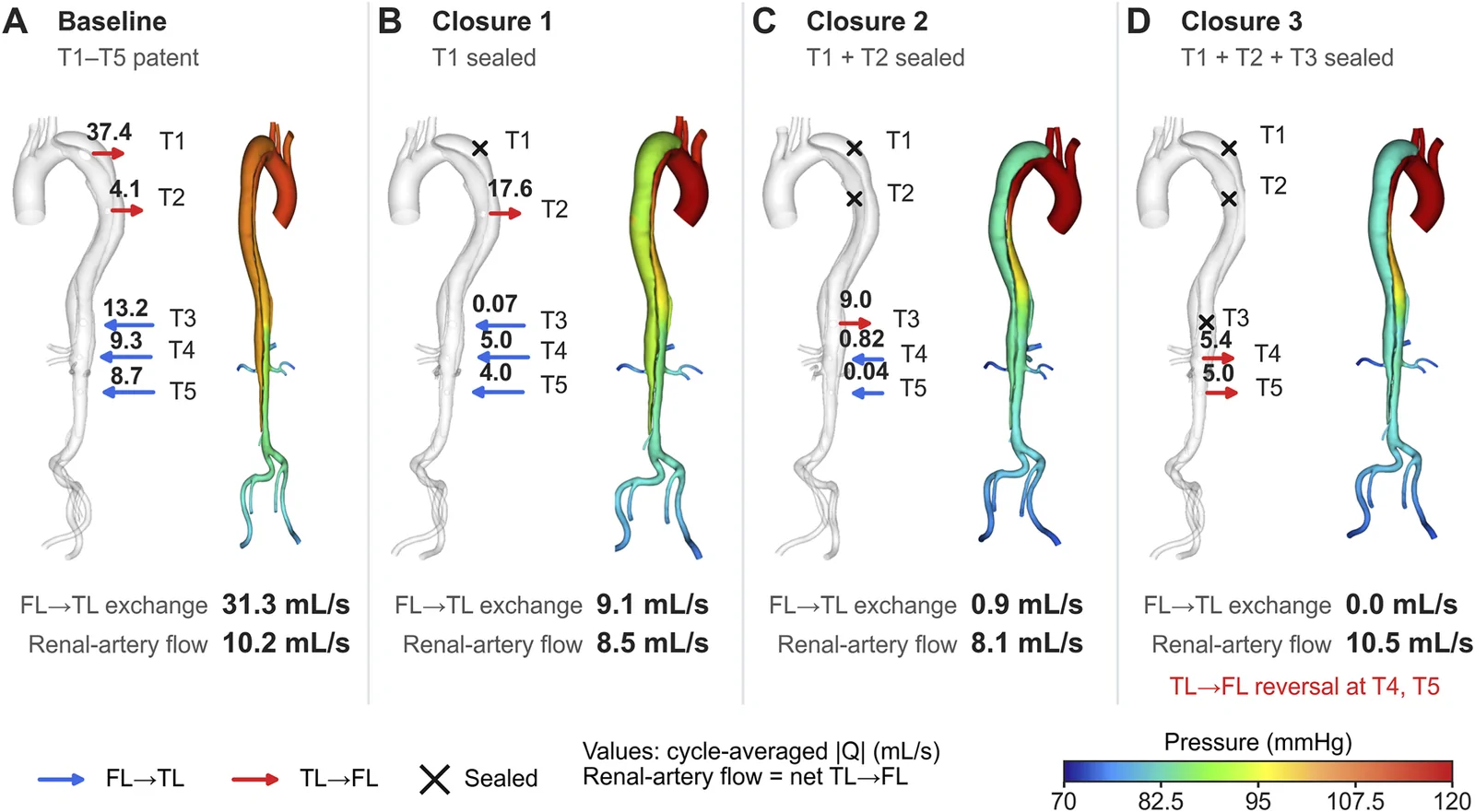
Virtual closure of idealized TL/FL communications in a patient-derived aortic geometry. Five communications (T1–T5) were created virtually in the preoperative aortic model of one atrophy case (RA-1) and do not correspond to that patient’s native tears. Four states were simulated under identical boundary and numerical settings: **(A)** baseline, **(B)** T1 sealed, **(C)** T1 + T2 sealed, and **(D)** T1 + T2 + T3 sealed. Values beside T1–T5 are cycle-averaged absolute trans-tear flow (mL/s), and arrows indicate direction (blue, FL→TL; red, TL→FL). The summed FL→TL exchange, which is directed toward the renal-ostial level, decreased from 31.3 to 9.1, 0.9 and 0.0 mL/s, while flow was redistributed among the remaining communications and T3, T4 and T5 successively reversed to TL→FL. Because the FL communicated only with the index renal artery, the net TL→FL transfer, shown below each panel, equals the index renal-artery flow (10.2, 8.5, 8.1 and 10.5 mL/s). Pressure maps share a common scale. The experiment is a conditional computational perturbation in a single model and is not a treatment recommendation.

## 4 Discussion

In this exploratory serial CTA-CFD study, with inlet and outlet boundary conditions held identical across time points, kidneys that later developed atrophy had a lower FL contribution to flow at the renal-ostial plane and lower total flow through residual communications than every comparator from the early postoperative model onward, whereas directly computed renal-artery flow and tear-exchange flow directed toward the renal-ostial level separated the groups only at late follow-up. These findings support assessment of residual communications by location, direction, and functional exchange rather than by patency alone.

Global renal function and unilateral volume loss diverged in this series. In one atrophy case, eGFR was 64.1 mL/min/1.73 m^2^ before repair and 64.7 at day 1,108, essentially unchanged, while the index kidney lost 53.0% of its volume; in a second, eGFR rose from 21.8 immediately before repair (52.0 at admission) to 61.1 at day 423, with a 64.1% loss of index-kidney volume. Preserved or recovering global function alongside progressive unilateral volume loss is compatible with a structural sequela of pre-repair malperfusion and illustrates why surveillance based on serum creatinine and eGFR, which reflect the combined function of both kidneys, may miss unilateral injury.

Because all six index kidneys were supplied from the FL before repair and all showed reduced parenchymal enhancement on preoperative CTA, neither luminal supply nor qualitative enhancement separated the two outcomes in this series. The hemodynamic analyses thus assessed functional differences within a similar anatomical pattern of FL renal supply.

At the renal-ostial aortic plane, velocity and FL flow-magnitude contribution changed more than the local pressure ratio. This pattern is physiologically plausible, because pressure can be transmitted through low-conductance pathways even when net exchange is small. However, the data do not establish a binary pressure-limited *versus* flow-limited mechanism; they are more consistent with a disproportionate reduction in local flow contribution relative to pressure change.

The FL flow-magnitude fraction describes the proportion of flow crossing the renal-ostial plane that is carried by the FL. FL flow is an established descriptor of FL perfusion in phase-contrast magnetic resonance studies of dissection, in which it has been used to characterize FL hemodynamics and has been associated with subsequent aortic growth (Clough et al., 2012; Burris et al., 2020). A low fraction with a preserved pressure ratio describes an FL that remains pressurized but carries little through-flow at the renal level, a low-washout condition that favors FL thrombosis (Menichini et al., 2018); FL thrombosis has in turn been associated with later renal atrophy (Yu et al., 2022). In the present series, the fraction in every atrophy kidney was lower than in every comparator by the early postoperative model, before the modeled renal-artery flow declined. We therefore hypothesize that early loss of FL through-flow at the level of an FL-dependent renal artery precedes, and may contribute to, later reduction in renal inflow; this hypothesis requires prospective testing.

Yang et al. (2026) examined renal-artery-to-aorta pressure ratios in three atrophy cases and proposed serial pressure assessment as an early indicator. The present analysis addresses a complementary question by including non-atrophic comparators and characterizing the distribution, direction, and aggregate flow of near-renal TL/FL communications.

Previous virtual-modification studies have shown that entry- or re-entry-tear manipulation can reverse inter-luminal flow direction and alter FL hemodynamics and pressure balance (Yu et al., 2016; Armour et al., 2020; Li et al., 2022; Motoki et al., 2023). These effects are established features of aortic dissection hemodynamics and are not specific to the present model.

In the present model, in which idealized communications were created within a patient-derived aortic geometry, sequential closure produced redistribution and reversal of flow within the residual communication network. After the upstream communications had been sealed, the communications near the renal-ostial level reversed from FL→TL to TL→FL, so that the FL at the renal level was no longer supplied by antegrade flow from above but only locally, while the net inter-luminal transfer, which in this model equals the index renal-artery flow, was similar after the third closure (10.5 mL/s) to that at baseline (10.2 mL/s). This pattern is consistent with the early postoperative finding in the patient models, in which exclusion of the entry tear was followed by a marked decrease in the FL flow-magnitude fraction at the renal-ostial plane. These results indicate that local TL/FL network organization is sensitive to tear configuration, and that sealing a larger number of communications is not necessarily associated with lower renal-artery flow, which in this model depended on the pressure balance between the two lumina and on the effective flow through the remaining communications.

Causality cannot be inferred from these data. Renal injury sustained before repair may independently influence both later structural loss and the downstream vascular resistance represented in the model. The timing of the differences is informative but not conclusive: the aortic-level flow measures separated the groups from the early postoperative model onward, whereas modeled renal-artery flow separated them only at late follow-up. In RA-2 and RA-3, the early separation preceded substantial volume loss, whereas in RA-1 the index kidney had already lost 36.9% of its volume by day 26 while its modeled renal-artery flow was unchanged (+2.7%). A sequence in which the FL flow contribution at the renal level decreases first and renal-artery flow later is compatible with an upstream hemodynamic contribution but does not establish one; with three kidneys per group, complete separation of a single measure is expected by chance in about one comparison in twenty, and 12 measures were examined. Because renal outlet impedance was held fixed, the modeled reductions cannot result from atrophy-related change in downstream resistance and reflect upstream geometric change; conversely, the model cannot show whether *in vivo* renal flow decreased further as renal mass was lost, or whether reduced renal run-off contributed to FL stasis. In one atrophy patient, the contralateral kidney also lost 15.3% of its volume, so late volume loss was not confined to the index side; however, the index kidney of that patient lost a further 35.3 percentage points, and no comparator showed a within-patient difference of similar magnitude. The findings are therefore associative and hypothesis-generating.

### 4.1 Limitations

This study has several limitations. First, the sample comprised six kidneys from six patients, and repeated tear-level and time-point observations nested within these patients do not increase the independent sample size. Although comparators were individually matched by age within 10 years and all participants were male, matching did not extend to repair strategy, renal inflow pattern, baseline renal function, or follow-up duration, and the comparator kidneys should not be regarded as physiologically normal controls. Repair strategy was nevertheless similar between groups, each containing one total arch replacement with frozen elephant trunk and two endovascular repairs. Aortic pathology was also heterogeneous: one patient had a type A intramural hematoma with distal dissection rather than a classical dissection. Selection depended on the availability of complete, reconstructable serial CTA and may have introduced selection bias.

Second, no patient had a directly measured pre-morbid renal baseline. In three patients, the first creatinine was drawn within 11 h of symptom onset and approximates pre-event function, and in one of these an independent value obtained 8 years earlier was normal; in two others, the first value was obtained 1–2 days after onset; no outpatient creatinine measurements predating admission were found in the hospital information system for the remaining patients. In the patient who presented 5 days after symptom onset, pre-event renal function could not be established. A pre-event eGFR of 64.1 mL/min/1.73 m^2^ in a patient with 20 years of hypertension is compatible with mild pre-existing nephrosclerosis.

Third, the CFD models assumed rigid walls and Newtonian blood, and used modeled inlet and outlet boundary conditions without direct calibration by 4D-flow MRI, Doppler, or invasive measurement. Fluid–structure interaction studies indicate that flap and wall motion can compress the distal true lumen by up to approximately 21% and shift the TL/FL flow split in acute unrepaired dissection (Chong et al., 2020), whereas in repaired type A dissection the TL–FL pressure difference is affected by less than 0.25 mmHg (Zhu et al., 2022). The present conclusions therefore do not depend on absolute wall-shear or pulse-pressure values, which are presented only as qualitative supporting evidence. In this cohort, the stented aortic segment was constrained by the graft and the flap was largely apposed, so wall mobility is expected to be lower than in acute untreated dissection. Patient-specific fluid–structure interaction modeling would itself be limited here, because flap thickness, elastic modulus and residual stress cannot be measured non-invasively from CTA, and such models necessarily adopt population-average material properties and a uniform wall thickness.

Fourth, renal outlet Windkessel parameters were estimated once, on the preoperative model, and held constant thereafter. This avoids circular dependence of the simulated renal flow on the renal-volume outcome but does not represent longitudinal change in downstream renal impedance. A formal sensitivity analysis of renal outlet resistance was not performed; the renal-flow results should therefore be interpreted as the hemodynamic consequences of geometric change under a fixed downstream-impedance assumption.

Fifth, communication direction and wall maps involved qualitative interpretation, and direction was classified by consensus without blinding to outcome group. The virtual closure experiment was performed in a single patient-derived geometry with virtually created communications; it does not represent the actual clinical situation and was not validated against clinical re-intervention. As endovascular techniques for blocking false-lumen backflow after thoracic endovascular aortic repair enter clinical use (Ge et al., 2024), the effect of closing distal communications on renal inflow should be examined in future studies. The early postoperative CTA was obtained between 3 and 37 days after repair, and in three patients the late CFD model preceded the final volumetric CTA, so hemodynamic and volumetric endpoints were not always contemporaneous. External validation is required in a larger cohort with standardized imaging intervals, three-dimensional renal volumetry, renal functional endpoints, and independent flow measurements.

## 5 Conclusion

In this exploratory study of six kidneys, with boundary conditions held identical across time points, kidneys that developed atrophy after aortic dissection repair had a lower FL flow contribution at the renal-ostial plane and lower total flow through residual communications than comparators from the early postoperative model onward; lower tear-exchange flow directed toward the renal-ostial level and a larger reduction in directly computed renal-artery flow followed at late follow-up. Local pressure ratios changed less and were heterogeneous. Because all six index kidneys were supplied from the FL before repair, luminal supply alone did not separate the two outcomes. These findings are associative and hypothesis-generating and should not be interpreted as proof of causality, as a measure of renal parenchymal perfusion, or as a basis for treatment decisions.

## Data Availability

The datasets presented in this article are not readily available because they contain identifiable patient imaging information; de-identified tabular data can be provided upon reasonable request and subject to institutional approval. Requests to access the datasets should be directed to Chao Yang, ychao@hust.edu.cn, or Yiqing Li, yiqingli_uh@126.com.
