# Supplementary material for: Serial computed tomography angiography-based computational fluid dynamics characterizes reduced near-renal tear exchange in kidneys that develop atrophy after aortic dissection repair
Source: Front Bioeng Biotechnol. 2026 Oct 29;14:1945232. doi: 10.3389/fbioe.2026.1945232 (PMC13754826; doi:10.3389/fbioe.2026.1945232)
Supplement: Supplementary file 1 [file Supplementaryfile1.docx]

**Supplementary Material**

Supplementary Table S1 (complete serial serum creatinine and eGFR by laboratory sampling time) is provided as a separate file.

**Supplementary Figure S1.** Mesh sensitivity: pressure over one cardiac cycle at the inlet and at eight outlets in one representative model, computed with maximum element sizes of 0.5 mm (reference; 5.519 million elements), 0.75 mm (2.307 million elements) and 1 mm (1.353 million elements). The values in each panel give the largest instantaneous difference relative to the 0.5-mm mesh.

**Supplementary Figure S2.** Mesh sensitivity: time-averaged wall shear stress (TAWSS), oscillatory shear index (OSI) and relative residence time (RRT) in the same model computed with the three meshes (maximum element sizes 0.5, 0.75 and 1 mm).

**Supplementary Figure S3.** Time-step sensitivity: pressure over one cardiac cycle at the inlet and at eight outlets in the same model, computed with fixed time steps of 0.001 s and 0.01 s and with adaptive time stepping at a Courant–Friedrichs–Lewy (CFL) number of 1.

**Supplementary Figure S4.** Time-step sensitivity: TAWSS, OSI and RRT in the same model for the three temporal settings (fixed 0.001 s, fixed 0.01 s and adaptive stepping with CFL = 1).
