# Supplementary material for: Serial computed tomography angiography-based computational fluid dynamics characterizes reduced near-renal tear exchange in kidneys that develop atrophy after aortic dissection repair
Source: Front Bioeng Biotechnol. 2026 Oct 29;14:1945232. doi: 10.3389/fbioe.2026.1945232 (PMC13754826; doi:10.3389/fbioe.2026.1945232)
Supplement: Supplementary file 2 [file Supplementaryfile2.docx]

**Supplementary Table S1. Complete serial serum creatinine and estimated GFR for all six patients, by time relative to repair**

Times are based on the laboratory sampling time recorded in the hospital information system (not the result-reporting time) and are expressed relative to the operative start time in the surgical record; calendar dates are not shown. eGFR was calculated with the CKD-EPI 2009 creatinine equation; values marked † were not reported in the source record and were calculated from the paired creatinine. Rows shaded green were sampled before repair; rows shaded red were sampled after repair.

| **Time relative to repair** | **Creatinine, µmol/L** | **eGFR, mL/min/1.73 m²** | **Note** |
| --- | --- | --- | --- |
| **RA-1 (age 53, male; repair duration 120 min)** | | | |
| pre-op −256.9 h | 112.3 | 64.1 | Last value before repair |
| post-op +11.3 h | 339.5 | 16.8 |  |
| post-op +64.7 h | 190.5 | 33.8 |  |
| post-op +593.8 h | 96.9 | 76.6 |  |
| post-op +8105.6 h | 94.8 | 78.1 |  |
| post-op +26585.8 h | 109.5 | 64.7 | Time point of the final volumetric measurement |
| **RA-2 (age 38, male; repair duration 50 min)** | | | |
| pre-op −276.2 h | 145.6 | 52.0 | Day after admission |
| pre-op −80.5 h | 510.9 | 11.4 |  |
| pre-op −54.4 h | 663.5 | 8.3 | Peak; K+ 6.82 mmol/L; CRRT initiated |
| pre-op −44.7 h | 582.7 | 9.7 |  |
| pre-op −33.9 h | 618.2 | 9.1 |  |
| pre-op −14.3 h | 299.4 | 21.8 | Last value before repair (after CRRT) |
| post-op +9.7 h | 235.2 | 29.1 |  |
| post-op +36.5 h | 259.6 | 25.8 |  |
| post-op +59.5 h | 232.0 | 29.6 |  |
| post-op +84.1 h | 217.8 | 31.9 |  |
| post-op +135.0 h | 175.9 | 41.4 |  |
| post-op +179.5 h | 149.4 | 50.4 |  |
| post-op +227.5 h | 137.8 | 55.6 |  |
| post-op +1673.0 h | 127.5 | 61.0 |  |
| post-op +5368.9 h | 121.3 | 64.4 |  |
| post-op +6376.6 h | 143.4 | 52.6 |  |
| post-op +7216.5 h | 141.4 | 53.5 |  |
| post-op +10148.9 h | 126.6 | 61.1 | Nearest to the final volumetric measurement |
| post-op +25697.1 h | 129.8 | 58.5 |  |
| **RA-3 (age 61, male; repair duration 336 min)** | | | |
| pre-op −258.4 h | 69.2 | 97.3 | Emergency department, before ward admission |
| pre-op −254.3 h | 66.8 | 98.7 | Last value before repair |
| post-op +16.1 h | 55.4 | 106.6 |  |
| post-op +65.6 h | 71.2 | 96.2 |  |
| post-op +139.4 h | 85.3 | 84.4 |  |
| post-op +211.2 h | 81.8 | 88.8 |  |
| post-op +283.7 h | 86.1 | 83.5 |  |
| post-op +2809.8 h | 115.9 | 58.3 |  |
| post-op +4152.4 h | 103.4 | 66.9 | Time point of the final volumetric measurement |
| post-op +8857.1 h | 117.5 | 56.9 |  |
| **non-RA-1 (age 51, male; repair duration 178 min)** | | | |
| pre-op −201.3 h | 83.9 | 92.4 | Emergency department, before ward admission |
| pre-op −174.1 h | 95.7 | 78.8 | Last value before repair |
| post-op +15.9 h | 85.8 | 90.0 |  |
| post-op +89.3 h | 75.2 | 100.8 |  |
| post-op +813.9 h | 96.9 | 77.6 |  |
| post-op +9045.1 h | 81.8 | 94.6 |  |
| post-op +17326.8 h | 82.8 | 92.6 | Time point of the final volumetric measurement |
| post-op +34677.6 h | 89.1 | 84.1 | Most recent follow-up |
| **non-RA-2 (age 55, male; repair duration 60 min)** | | | |
| pre-op, day of admission (5 days before repair; time not recorded) | 75.0 | 98.2 † | Day of admission (value from the discharge summary; sampling time not recorded); only pre-operative value |
| post-op +12.1 h | 76.3 | 97.5 † |  |
| post-op +66.3 h | 81.5 | 93.1 † |  |
| post-op +132.8 h | 78.0 | 96.6 † |  |
| **non-RA-3 (age 46, male; repair duration 308 min)** | | | |
| pre-op −75414.9 h | 69.2 | 108.1 † | Eight years before the aortic event |
| pre-op −322.5 h | 88.8 | 89.4 | Admission |
| pre-op −58.6 h | 74.9 | 104.6 | Last value before repair |
| post-op +12.1 h | 52.1 | 121.5 |  |
| post-op +36.1 h | 68.9 | 108.3 |  |
| post-op +109.4 h | 61.5 | 113.5 |  |
| post-op +181.8 h | 72.4 | 106.1 |  |
| post-op +229.3 h | 80.8 | 100.2 |  |
| post-op +593.7 h | 89.3 | 88.1 |  |
| post-op +1266.0 h | 75.1 | 103.8 |  |
| post-op +4122.1 h | 87.3 | 90.6 |  |
| post-op +8490.8 h | 89.3 | 88.1 | Nearest to the final volumetric measurement |
| post-op +17058.4 h | 85.6 | 92.7 |  |
| post-op +18455.0 h | 74.1 | 103.1 | Most recent follow-up |
